# Alterations of long-range association fibers in patients with anti-N-methyl-D-aspartate receptor encephalitis

**DOI:** 10.1016/j.nicl.2025.103808

**Published:** 2025-05-24

**Authors:** Xiaodong Chen, Ling Fang, Yiying Huang, Yu Huang, Yi Lu, Jinhui Wang, Chunxin Liu, Huanquan Liao, Liemin Zhou, Wei Qiu, Yaqing Shu

**Affiliations:** a Department of Neurology, The Seventh Affiliated Hospital, Sun Yat-sen University, No. 628, Zhenyuan Road, Guangming District, Shenzhen 518106 Guangdong, China; b Department of Neurology, The Third Affiliated Hospital of Sun Yat-sen University, No. 600, Tianhe Road, Tianhe District, Guangzhou 510630 Guangdong, China; c Department of Radiology, The Third Affiliated Hospital of Sun Yat-sen University, No. 600, Tianhe Road, Tianhe District, Guangzhou 510630 Guangdong, China; d Department of Neurology, The Eighth Affiliated Hospital of Sun Yat-sen University, No. 3025, Shennan Road, Futian District, Shenzhen 518000 Guangdong, China; e Institute for Brain Research and Rehabilitation, Guangdong Key Laboratory of Mental Health and Cognitive Science, Center for Studies of Psychological Application, South China Normal University, Guangzhou 510630, China; f Department of Emergency, The Third Affiliated Hospital of Sun Yat-sen University, No. 600, Tianhe Road, Tianhe District, Guangzhou 510630 Guangdong, China

**Keywords:** Anti-N-methyl-d-aspartate receptor encephalitis, Diffusion tensor imaging, Long-range association fiber, Mean diffusivity, Radial diffusivity, Cognition

## Abstract

**Background:**

Patients with anti-NMDAR encephalitis typically exhibit impaired cognitive integration, which relies on the integrity of long-range association fibers connecting diverse brain regions. However, the microstructural integrity of long-range association fibers in this population remains unknown.

**Methods:**

Diffusion tensor imaging (DTI) data were collected from 32 patients with anti-NMDAR encephalitis and 30 healthy controls. Patients were further categorized into early and delayed immunotherapy subgroups based on a 2-week threshold for immunotherapy initiation. The diffusion properties of major long-range association fibers were quantified at both the bundle and node levels.

**Results:**

Compared with healthy controls, patients exhibited widespread microstructural damage within long-range association fibers, with more severe alterations in the delayed immunotherapy subgroup (FDR-corrected *p* < 0.05). In this subgroup(n = 14), radial diffusivity (RD) of left inferior fronto-occipital fasciculus (IFOF), left inferior longitudinal fasciculus (ILF), left superior longitudinal fascicles (SLF), and bilateral arcuate fascicles correlated significantly with global cognition (MMSE, FDR-corrected *p* < 0.05). Notably, RD also strongly correlated with working memory in the delayed immunotherapy subgroup, showing bundle-wise associations for IFOF (left: *r =* -0.8315, *p =* 0.0112; right: *r =* -0.7044, *p =* 0.0295), ILF (left: *r =* -0.7473, *p =* 0.0243), SLF (left: *r =* -0.7562, *p =* 0.0243; right*: r =* -0.6599, *p =* 0.0391), and arcuate fasciculus (left: *r =* -0.7240, *p =* 0.0272; right: *r =* -0.6835, *p =* 0.0333), with left-hemisphere predominance confirmed by node-wise analyses of IFOF, ILF, SLF, and arcuate fasciculus (FDR-corrected *p* < 0.05).

**Conclusions:**

Our findings highlight widespread microstructural damage in long-range association fibers in patients with anti-NMDAR encephalitis, particularly in those with delayed immunotherapy. This damage may serve as the neurophysiological basis for cognitive impairments, with working memory being most affected.

## Highlights

• Microstructural damage of long-range association fibers marks anti-NMDAR encephalitis despite normal MRI.
• Delayed immunotherapy exacerbates long-range association fiber damage in patients with anti-NMDAR encephalitis.
• Compromised integrity of long-range association fibers links to cognitive decline, especially working memory.

## 1 Introduction

Anti-N-methyl-D-aspartate receptor (anti-NMDAR) encephalitis is a rare and severe autoimmune encephalitis characterized by rapidly progressive psychiatric symptoms, seizures, cognitive impairment, movement disorders, decreased consciousness, and autonomic dysfunction(Dalmau et al., 2008). While traditionally thought to primarily target neurons with minimal white matter involvement(Chen et al., 2023, Finke et al., 2013, Titulaer et al., 2013), recent diffusion MRI studies have highlighted white matter damage as an important component of its pathophysiology (Finke et al., 2013, Phillips et al., 2018, Yang et al., 2022). White matter association fibers, which are critical for effective neurotransmission and interregional communication, can be categorized into short association fibers (U-fibers) and long-range association fibers. Previous research has linked U-fiber damage to clinical cognitive impairments in anti-NMDAR encephalitis patients with incomplete recovery(Phillips et al., 2018). However, little is known about the microstructural integrity of long-range association fibers, which are crucial for multimodal information integration across distant cortical regions (Dou et al., 2020).

Diffusion tensor imaging (DTI) studies using tract-based spatial statistics have consistently reported microstructural damage within the deep white matter(Finke et al., 2013, Liang et al., 2020, Liu et al., 2022, Wang et al., 2024, Wei et al., 2022, Yang et al., 2022). However, these studies typically focus on global white matter changes and fail to address the specific involvement of long-range association fibers, which are critical for long-range connectivity. Mechanistically, recent evidence suggests that NMDAR-IgG-mediated neuroinflammation impedes myelination and remyelination by affecting oligodendrocyte NMDAR signaling (Matute et al., 2020). The oligodendrocyte NMDAR signaling pathway regulates the supply of energy substrates via glucose transporter translocation into the myelin compartment, bolstering energy support for fast-spiking axons (Saab et al., 2016). Given their substantial energy demands, long-range association fibers may be particularly vulnerable to such disruptions in patients with anti-NMDAR encephalitis(Ju et al., 2016). Furthermore, clinical studies have identified delayed immunotherapy as a major predictor of poor outcomes in these patients (Balu et al., 2019, Titulaer et al., 2013). However, its potential role in contributing to microstructural damage within long-range association fibers and its implications for cognitive outcomes remain unclear.

In the present study, we analyzed the diffusion properties of major long-range association fibers at bundle- and node-wise levels. The primary objectives were to (i) identify subtle microstructural damage in long-range association fibers in patients with anti-NMDAR encephalitis, and (ii) investigate the distinctive patterns of fiber involvement in subgroups stratified by the timing of immunotherapy initiation, as well as their associations with cognitive performance.

## 2 Methods

### 2.1 Study participants

**Fig. 1:**
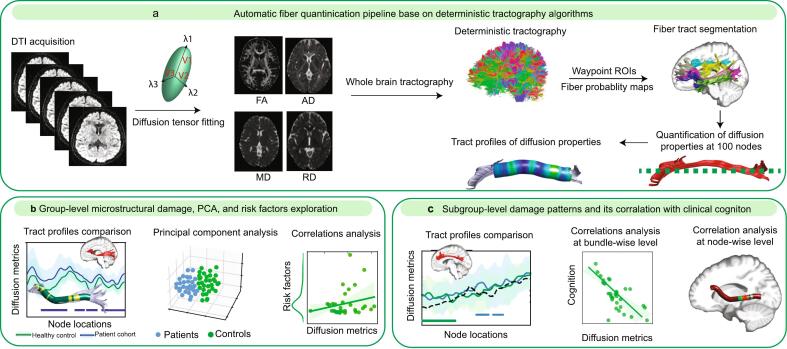
Sketch of the study framework. **a)** Automated fiber quantification of long-range association fibers based on the deterministic tractography algorithm. **b)** Group-level diffusion metrics comparisons, principal component analysis of diffusion metrics and exploration of risk factors correlated with long-range association fibers damage. **c)** Subgroup-level tract profiles of diffusion metrics within long-range association fibers and association with cognitive performance.

Thirty-four patients with anti-NMDAR encephalitis were recruited consecutively at The Third Affiliated Hospital of Sun Yat-sen University in southern China between March 2019 and March 2023. All patients were diagnosed with definite anti-NMDAR encephalitis based on the clinical criteria for anti-NMDAR encephalitis (Graus et al., 2016). All DTI neuroimaging and cognitive assessments were conducted during the convalescent phase (≥3 months post-onset), when patients achieved neurological stability assessed by the modified Rankin scale (mRS 0–3). The clinical symptomatology, laboratory examinations, and structural MRI during disease onset were also retrieved from the electronic medical records system. Patients who met the following criteria were excluded from the present study: (i) had autoantibodies suggestive of other autoimmune encephalitis or paraneoplastic syndromes; (ii) had remarkable structural lesions evident in T1-weighted or FLAIR imaging; and (iii) had DTI data affected by visible artifacts or head motion. A series of 31 neurologically healthy individuals were recruited as healthy controls in the study. Ethical approval was granted by the Medical Ethics Committee of the Third Affiliated Hospital of Sun Yat-sen University in Guangdong Province, China [(2019)2–637], and carried out in accordance with the principles described in the Declaration of Helsinki. Written informed consents were obtained from all participants. The study framework is shown in Fig. 1.

### 2.2 Clinical dataset collection

The cerebrospinal fluid (CSF) and serum NMDAR-IgG levels were assessed using a cell-based transfected immunofluorescence assay as previously described (Shu et al., 2019). Neuropsychological assessments, including Mini-mental State Examination (MMSE) and Montreal Cognitive Assessment (MoCA), were conducted by a single trained neuropsychologist (blinded to MRI findings) ≥ 3 months post-onset and ≥ 1 month after first-line immunotherapy, using standardized scales to ensure evaluation. The electroencephalography features indicative of encephalitis included focal or diffuse slow activity, epileptic activity, or the presence of an extreme delta brush. Positive MRI was defined by features suggestive of autoimmune encephalitis, including T2 fluid attenuated inversion recovery (T2-FLAIR) hyperintensities in the medial temporal lobes or multifocal inflammatory lesions in grey matter and white matter (Graus et al., 2016), but was not used as a diagnostic requirement for anti-NMDAR encephalitis. All patients were screened for teratomas using ultrasonic examination or pelvic MRI. The second-line immunotherapy included intravenous cyclophosphamide and B-cell depletion induced by rituximab.

### 2.3 MRI acquisition

MRI was conducted on a 3.0Tesla MR scanner (Discovery MR750, General Electric Medical Systems, Milwaukee, Wisconsin, United States) in the Department of Radiology. MRI sequences were acquired with the following parameters: (i) high-resolution three-dimensional fast spoiled gradient echo-brain volume (3D-FSPGR BRAVO) T1-weighted imaging: repetition time (TR) = 8.2 ms, echo time (TE) = 3.2 ms, field of view (FOV) = 256 × 256  mm, matrix = 256 × 256, flip angle (FA) = 12°, voxel size = 1 × 1 × 1 mm, slice thickness = 1 mm; (ii) three-dimensional CUBE T2-FLAIR-weighted imaging: TR = 6000 ms, TE = 130 ms, inversion time (TI) = 1843 ms, bandwidth = 41.67 kHz, FA = 145°, matrix = 256 × 256, FOV = 256 × 256 mm, slice thickness = 1 mm, and number of excitations = 1; and (iii) single-shot spin-echo echo-planar imaging sequence for diffusion tensor imaging: T*R =* 8500 ms, TE = 60.5 ms, 70 axial slices, voxel size = 2 × 2 × 2 mm, matrix = 128 × 128, FOV = 256 × 256 mm, slice thickness = 2 mm. Diffusion gradients were applied in 55 gradient directions (b-value = 1000 s/mm^2^), and one dataset was acquired with no diffusion weighting (b-value = 0 s/mm^2^). Foam padding was used to fix the head to minimize head motion, and all participants were instructed to remain as motionless as possible during the scan.

### 2.4 Diffusion tensor image preprocessing and analysis

The DTI datasets were preprocessed using the FMRIB Diffusion Toolbox (FDT), a module of the FMRIB Software Library (FSL, Department of Clinical Neurology, University of Oxford, Oxford, United Kingdom; https://www.fmrib.ox.ac.uk/fsl). The preprocessing steps included correction for eddy current artifacts and head motion, followed by adjustment of the diffusion gradient tables to account for eddy current correction. Individualized brain masks were generated from the volumes with b-values = 0. Diffusion tensor metrics, including fractional anisotropy (FA), mean diffusivity (MD), radial diffusivity (RD), and axial diffusivity (AD), were computed by fitting a tensor model to the preprocessed diffusion data within the individualized brain masks. Fourteen major long-range association fibers (listed in Supplementary Table S1), including the bilateral cingulum cingulate component, cingulum hippocampal component, inferior fronto-occipital fascicles (IFOF), inferior longitudinal fascicles (ILF), superior longitudinal fascicles (SLF), uncinate fascicles, and arcuate fascicles, were reconstructed using the automated fiber quantification pipeline (https://github.com/jyeatman/AFQ)(Yeatman et al., 2012) implemented in MATLAB R2020b (MathWorks, Inc., Natick, Massachusetts, USA). Briefly, whole-brain fiber tractography was performed using a deterministic streamline tractography algorithm based on preprocessed diffusion tensor metrics. Tracking was terminated if the FA dropped below 0.2 or if the minimum angle between the current path and the next step direction exceeded 30 degrees. Fiber tract segmentation was achieved by applying waypoint region of interest (ROI) procedures to isolate the central portions of each tract, where fibers were most coherently bundled (Wakana et al., 2007). The ROI definitions were adapted from standardized templates in MNI space and transformed to individual native space(Hua et al., 2008). Refinement of fiber tracts was based on probabilistic fiber tract atlases to ensure anatomical consistency. Fibers deviating more than four standard deviations above the mean fiber length or five standard deviations from the core of the fiber tract were excluded. The cleaned and segmented fiber tracts were resampled to 100 equidistant segments to generate tract profiles. Diffusion metrics (e.g., FA, MD, RD, AD) were then computed along the central portion of each tract using spline interpolation. These profiles enable the quantification of diffusion properties at anatomically equivalent locations along each tract.

### 2.5 Statistical analysis

No sample size calculations were performed in this retrospective observational study, and the sample size of the present study was determined based on recent diffusion MRI studies(Liang et al., 2020, Liu et al., 2022, Phillips et al., 2018, Yang et al., 2022). The statistical analysis was conducted using *R* (version 4.1.2, R Foundation for Statistical Computing, Vienna, Austria). For demographic and clinical variables, Shapiro-Wilk normality tests were performed to assess the normality of the distribution of continuous measurement data. The descriptive results were reported as the mean ± standard deviation for normally distributed variables or as the median (interquartile range, IQR) for nonnormally distributed variables. Univariate analyses were performed using either Student’s *t* tests for normally distributed data or Mann-Whitney U tests for nonnormally distributed data. Categorical variables were compared using Pearson’s chi-squared tests. A two-tailed *p* value of less than 0.05 was considered to indicate statistical significance.

Analysis of covariance (ANCOVA) was performed to compare diffusion properties (FA, MD, RD, and AD) within each long-range association fiber at bundle-wise and node-wise level, controlling for the effects of sex and age. At the bundle-wise level, we compared the average diffusion properties of the entire fiber tract. At node-wise level, we examined the diffusion properties of individual nodes within the fiber tract. Both the bundle-wise and node-wise diffusion property analyses were adjusted by false discovery rate (FDR) multiple comparison correction. Only those segments comprising more than five adjacent nodes with adjusted significant *p* values (FDR-corrected *p* value < 0.05) are reported in the results. Principal component analysis (PCA) was independently conducted for FA, MD and RD metrics. The input data structure was “62 subjects × 14 fiber bundles per metric (FA/MD/RD). For each diffusion metric, mean bundle-wise values from 14 predefined long-range fiber bundles across all subjects were z-score standardized, followed by covariance matrix decomposition with a prespecified 3-component solution (number of components = 3).

Patients were stratified into early-immunotherapy (≤2 weeks post-onset) and delayed-immunotherapy (>2 weeks) subgroups. To delineate subgroup-specific patterns of long-range association fiber degeneration, we performed ANCOVA models comparing bundle-wise and node-wise diffusion metrics among healthy controls, early immunotherapy, and delayed immunotherapy subgroups, with age and sex as covariates. Post-hoc pairwise comparisons were conducted where significant main effects emerged. In parallel, partial correlation analyses adjusted for age and sex examined relationships between altered diffusion metrics and cognitive scores, with statistical significance determined by FDR-corrected *p*-values < 0.05.

## 3 Results

### 3.1 Clinical characteristics

Two patients and one healthy control with significant head motion were excluded from the subsequent analysis. The analyses included a total of 32 patients and 30 healthy controls. There was no significant difference between the patient cohort and healthy controls regarding age (26.59 ± 11.11 years vs. 29.57 ± 5.11 years, *df* = 60, *t* = 1.339, *p =* 0.1858) or female predominance (65.6 % vs. 63.3 %, χ^2^ = 0.0*, p* > 0.9999). The median interval from disease onset to MRI acquisition was 8.79 (IQR, 6.07–29.77) months. Among 32 patients, 7 (21.9 %) exhibited acute-phase MRI abnormalities meeting autoimmune encephalitis criteria: 4 with isolated medial temporal lobe hyperintensity and 3 with multifocal inflammatory lesions. Notably, all acute-phase MRI abnormalities had resolved completely by the convalescent phase following immunotherapy, with no persistent radiographic abnormalities observed at study enrollment. The clinical profiles of the patient cohort are presented in Table 1.

**Table 1:** Clinical profiles of the patients with anti-NMDAR encephalitis.

Clinical variables	Patient cohort (N = 32)
Age, years	26.59 ± 11.11
Female, %	65.6 %
Interval from onset to MRI acquisition	8.79 (IQR, 6.07–29.77)
Teratoma at disease onset, %	25.0 %
Onset CSF NMDAR-IgG titter	1:30 (IQR, 1:6.6–1:100)
Onset mRS	5.0 (IQR, 4.0–5.0)
Convalescent mRS	1.5 (IQR, 0.0–2.0)
Symptomatology, onset; convalescent	
Psychiatric symptoms	96.9 %; 34.4 %
Seizure	81.3 %; 9.4 %
Reduced consciousness	46.9 %; 0.0 %
Movement disorder	50 %; 0.0 %
MMSE score	27.2 ± 2.7
Abnormal EEG at disease onset, %	92.3 %
Positive MRI^a^ at disease onset, %	21.9 %
Positive MRI during convalescence, %	0.0 %
Immunotherapy during convalescence	
Corticosteroids, n, %	8 (25.0 %)
Mycophenolate mofetil (MMF), n, %	2 (6.25 %)
Corticosteroids + MMF	8 (25.0 %)
Rituximab, n, %	14 (43.75 %)

a : Positive MRI findings indicated the features suggestive of encephalitis, including T2-FLAIR hyperintensities in medial temporal lobes or multifocal inflammatory lesions in grey matter and white matter. Abbreviations: IQR, interquartile range; anti-NMDAR, anti-N-methyl-D-aspartic acid receptor; MRI, magnetic resonance imaging; CSF, cerebrospinal fluid; IgG, immunoglobulin G; mRS, modified Rankin scale; MMSE, mini-mental state examination; EEG, electroencephalograph; MMF, mycophenolate mofetil.

### 3.2 Bundle-wise diffusion metrics alterations

**Fig. 2:**
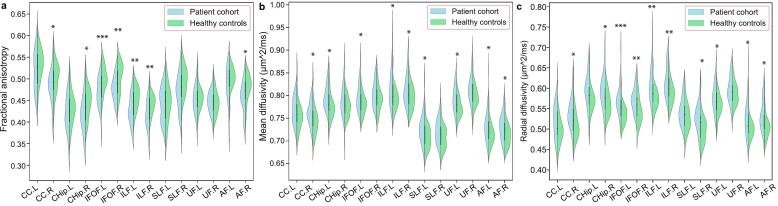
Group differences of bundle-wise diffusion properties within long-range association fibers. (**a)** Group comparison of bundle-wise mean fractional anisotropy (FA), (**b**) mean diffusivity (MD) and **(c)** radial diffusivity (RD) between healthy controls and the patient cohort (FDR-corrected *p <* 0.05, adjusted for sex and age, * *p* < 0.05, ** *p* < 0.01, *** *p* < 0.001). Abbreviations: CC, cingulum cingulate component; CHip, cingulum hippocampal component; IFOF, inferior fronto-occipital fasciculus; ILF, inferior longitudinal fasciculus; SLF, superior longitudinal fasciculus; UF, uncinate fasciculus; AF, arcuate fasciculus; FA, fractional anisotropy; MD, mean diffusivity; RD, radial diffusivity.

Bundle-wise DTI analyses demonstrated significant microstructural alterations in patients compared to healthy controls (Fig. 2, Supplementary Table S2). Specifically, FA was reduced in the right cingulum (including both cingulate and hippocampal components), bilateral IFOF, bilateral ILF, and right arcuate fasciculus. MD showed significant elevation in the right cingulum cingulate component, left cingulum hippocampal component, left IFOF, bilateral ILF, left SLF, left uncinate fasciculus, and bilateral arcuate fascicles. Furthermore, RD was markedly increased in the right cingulum (both cingulate and hippocampal components), bilateral IFOF/ILF/arcuate fasciculus, right SLF, and left uncinate fasciculus.

### 3.3 Node-wise tract profiles of diffusion metrics

**Fig. 3:**
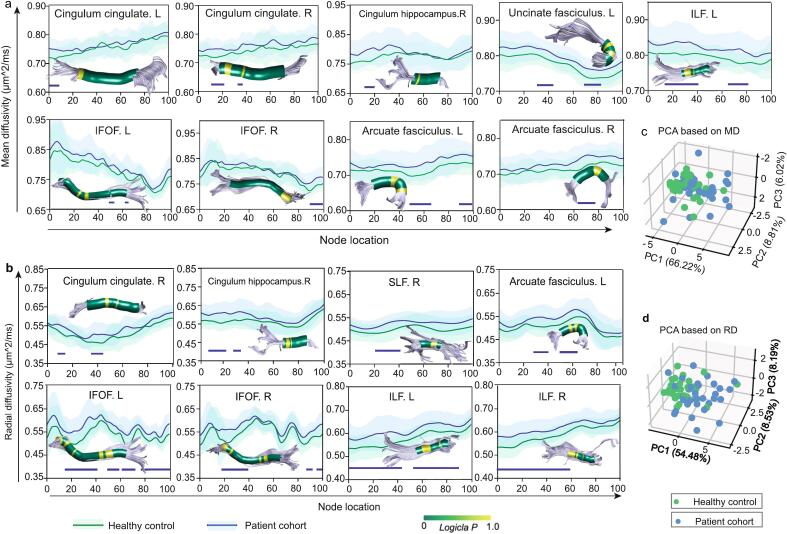
Group differences in node-wise diffusion metrics and principal component analysis (PCA) based on bundle-wise diffusion metrics. (**a**) Group differences in node-wise mean diffusivity (MD) and (**b**) radial diffusivity (RD) of major long-range association fibers. The node-wise tract profiles were rendered in the fascicles, with yellow segments indicating subregions showing statistically significant higher MD or RD in the patient cohort after FDR correction (< 0.05), adjusted for sex and age. (**c**) Three-dimensional PCA based on bundle-wise MD and (**d**) RD of long-range association fiber. Abbreviations: IFOF, inferior fronto-occipital fasciculus; ILF, inferior longitudinal fasciculus; SLF, superior longitudinal fasciculus; RD, radial diffusivity; MD, mean diffusivity; PCA, principal component analysis; PC, principal component. (For interpretation of the references to colour in this figure legend, the reader is referred to the web version of this article.)

MD and RD analyses revealed extensive microstructural alterations in patients versus controls across major long-range association fibers (all FDR-corrected *p* < 0.05, age and sex-adjusted, Fig. 3a and 3b): left cingulum cingulate component (MD: nodes 1–8) and right cingulum cingulate component (MD: nodes 11–21; RD: nodes 10–16 and 36–47); right cingulum hippocampal component (MD: nodes 13–20; RD: nodes 9–24 and 29–33); left IFOF (MD: nodes 52–56; RD: nodes 17–41, 51–58, 63–72, and 78–100) and right IFOF (MD: nodes 89–99; RD: nodes 19–39, 85–91, and 94–100); left ILF (MD: nodes 14–41 and 65–82; RD: nodes 1–43 and 52–89) and right ILF (RD: nodes 1–59); left uncinate fasciculus (MD: nodes 33–45 and 67–82); left arcuate fasciculus (MD: nodes 48–69 and 88–100; RD: nodes 27–39 and 49–65) and right arcuate fasciculus (MD: nodes 63–77); right SLF(RD: nodes 23–41). The node-wise FA tract profiles of major long-range association fibers were presented in Supplementary Fig. S1a. In our study, there was no significant difference in AD between healthy controls and patients with anti-NMDAR encephalitis at either the bundle-wise or node-wise level.

### 3.4 Principal component analysis of bundle-wise diffusion metrics

Three-dimensional PCA revealed that MD and RD collectively captured 81.05 % and 71.20 % of the variance in microstructural alterations, respectively, outperforming FA (58.69 %, Supplementary Fig. S1b) in discriminating patient-control differences (Fig. 3c-d). The loadings of each principal component are presented in Supplementary Tables S3–S5. Given this superior sensitivity of MD and RD to disease-related changes, all subsequent analyses focused on these two metrics to investigate immunotherapy timing effects and cognition-structure relationships.

### 3.5 The microstructural alteration of long-range association fibers in subgroups stratified by the timing of immunotherapy initiation

**Fig. 4:**
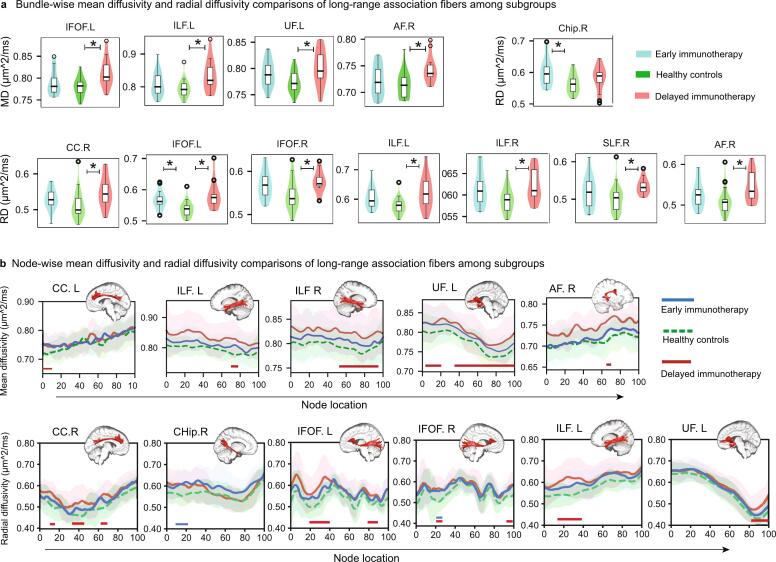
Bundle-wise and node-wise comparisons of mean diffusivity (MD) and radial diffusivity (RD) among healthy controls, the early immunotherapy subgroup, and the delayed immunotherapy subgroup. (**a)** Bundle-wise MD and RD comparisons at the subgroup level (FDR-corrected *p* < 0.05, adjusted for sex and age, *: *p* < 0.05, ** *p* < 0.01). (**b)** Node-wise MD and RD comparisons at the subgroup level (FDR-corrected *p* < 0.05, adjusted for sex and age). Abbreviations: MD, mean diffusivity; RD, radial diffusivity; CC, cingulum cingulate component; CHip, cingulum hippocampal component; IFOF, inferior fronto-occipital fasciculus; ILF, inferior longitudinal fasciculus; SLF, superior longitudinal fasciculus; UF, uncinate fasciculus; AF, arcuate fasciculus.

Interestingly, the interval from disease onset to first-line immunotherapy initiation (days) correlated with elevated MD/RD of multiple long-range association fibers (Supplementary Fig. S2). To assess the clinical relevance of these timing-effects, patients were stratified by immunotherapy initiation (early ≤ 2 weeks vs. delayed > 2 weeks post-onset). The clinical characteristics of the two subgroups are summarized in Table 2. Bundle-wise analyses revealed significantly elevated MD and RD of most long-range association fibers in the delayed immunotherapy subgroup versus healthy controls (FDR-corrected *p <* 0.05, age and sex-adjusted; Fig. 4a, Supplementary Table S6). Node-wise profiles demonstrated extensive microstructural damage in this subgroup. MD increases were observed in the left cingulum cingulate component (nodes 1–10), bilateral ILF (left nodes 72–78; right nodes 49–95), left uncinate fasciculus (nodes 4–20 and 35–100), and right arcuate fasciculus (nodes 65–70). For RD, significant increases were identified in the right cingulum cingulate component (nodes 11–15, 37–47, and 63–69), bilateral IFOF (left nodes 19–40, and 81–87; right nodes 22–28 and 95–99), left ILF (nodes 16–38), and left uncinate fasciculus (nodes 85–100). In contrast, the early immunotherapy subgroup showed limited damage, with only the right cingulum hippocampal component (nodes 13–22) and right IFOF (node 22–28) affected (FDR-corrected *p <* 0.05, age and sex-adjusted, Fig. 4b). Notably, the delayed subgroup exhibited unique RD elevation in the left uncinate fasciculus (nodes 92–100) compared to early subgroup.

**Table 2:** Clinical characteristics of patient subgroups stratified by the timing of immunotherapy initiation.

Clinical variables	Early immunotherapy subgroup (N = 18)	Delayed immunotherapy subgroup(N = 14)	*p* value
Age, years	22.0 (IQR,17.8–30.3)	26.5(IQR,19.8–38.3)	0.2023
Female, %	66.67 %	57.14 %	0.8540
Interval from onset to DTI acquisition, months	7.4 (IQR, 4.7–14.1)	23.4 (IQR, 8.7–43.9)	**0.0115**
Teratoma, %	33.33 %	14.29 %	0.4105
CSF WBC (cells/μl)	2.0 (IQR, 0.0–6.0)	2.0 (IQR, 0.0–6.5)	0.7392
mRS	1.0 (IQR, 0.0–2.0)	2.0 (IQR, 3.0–5.0)	0.6444
Symptomatology			
Psychiatric symptoms	38.89 %	28.57 %	0.8146
Seizure	11.11 %	7.14 %	1
Reduced consciousness	0	0	/
Movement disorder	0	0	/
MRI suggestive of encephalitis, %	0	0	/
MMSE score	27.33 ± 2.59	27.07 ± 3.00	0.7754
Mechanical ventilation at disease onset	61.11 %	21.43 %	0.0593
ICU admission at disease onset, %	66.67 %	28.50 %	0.0748
Time to immunotherapy initiation, days	7.0 (IQR, 5.0–8.5)	23.5(IQR, 20.0–60.3)	**＜0.0001**
Second-line immunotherapy, %	44.44 %	50.00 %	1

Abbreviations: IQR, interquartile range; DTI, diffusion tensor imaging; CSF, cerebrospinal fluid; WBC, white blood cells; IgG, immunoglobulin G; mRS, modified Rankin scale; MRI, magnetic resonance imaging; MMSE, mini-mental state examination; ICU, intensive care unit.

### 3.6 Correlation between diffusion metrics of long-range association fibers and cognition

**Fig. 5:**
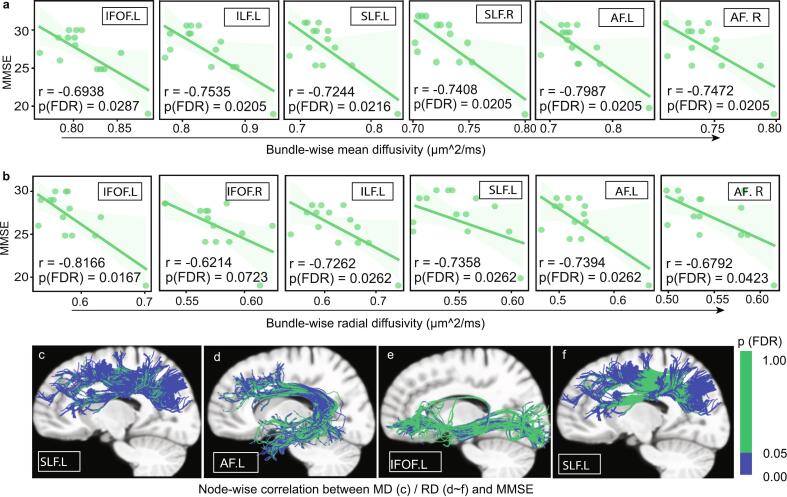
Correlation analyses between diffusion metrics of long-range association fibers and cognition in the delayed immunotherapy subgroup. (**a)** Bundle-wise correlation between mean diffusivity (MD) / (**b**) radial diffusivity (RD) of long-range association fibers and Mini-Mental State Examination (MMSE) score. (**c ∼ f**) Node-wise correlation between diffusion metrics of affected long-range association fibers and MMSE scores (**c**: left superior longitudinal fasciculus (SLF) MD; **d:** left arcuate fasciculus RD; **e:** left inferior fronto-occipital fasciculus RD; **f**: left SLF RD). All analyses are FDR-corrected (*p* < 0.05) and adjusted for sex and age. Abbreviations: MMSE, mini-mental state examination; IFOF, inferior fronto-occipital fasciculus; ILF, inferior longitudinal fasciculus; SLF, superior longitudinal fasciculus; UF, uncinate fasciculus; AF, arcuate fasciculus.

In the delayed immunotherapy subgroup (n = 14), adjusted analyses revealed significant negative correlations between mean MD/RD values of major long-range association fibers and cognitive performance. For global cognition, MMSE scores demonstrated strong associations with bundle-wise metrics: left IFOF (MD: *r =* -0.6938, *p =* 0.0287; RD: *r =* -0.8166, *p =* 0.0167), left ILF (MD: *r =* -0.7535, *p =* 0.0205; RD: *r =* -0.7262, *p =* 0.0262), bilateral SLF (left MD: *r =* -0.7244, *p =* 0.0216, RD: *r =* -0.7358, *p =* 0.0262; right MD: *r =* -0.7408, *p =* 0.0205), and bilateral arcuate fasciculus (left MD: *r =* -0.7987, *p =* 0.0205, RD: *r =* -0.7394, *p =* 0.0262; right MD: *r =* -0.7472, *p =* 0.0205, RD: *r =* -0.6792, *p =* 0.0423) (FDR-corrected *p <* 0.05, age and sex-adjusted, Fig. 5a-b).The Node-wise correlation analyses between diffusion metrics and MMSE revealed significant segments within left SLF (MD, nodes 1–14 and 32–100; RD, nodes1-16 and 78–100), left IFOF (RD, nodes 1–21 and 34–46), and left arcuate fasciculus (RD, nodes 1–21 and 36–68) (FDR-corrected *p <* 0.05, age and sex-adjusted, Fig. 5c ∼ f).

**Fig. 6:**
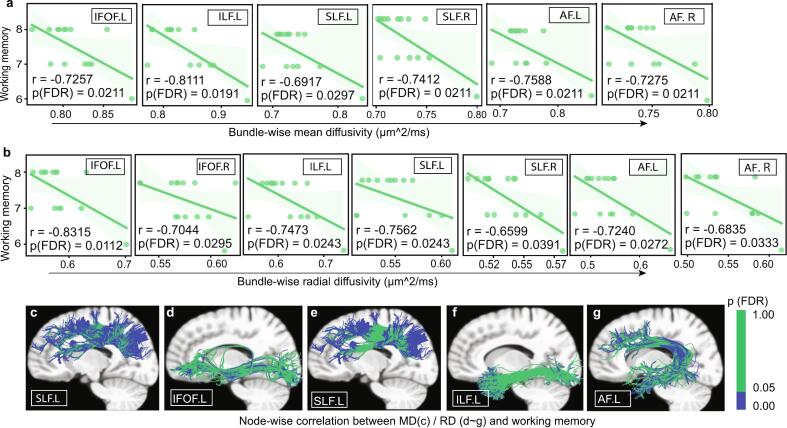
Correlation analyses between diffusion metrics of long-range association fibers and working memory in the delayed immunotherapy subgroup. (**a)** Bundle-wise correlation between mean diffusivity (MD) / (**b**) radial diffusivity (RD) of long-range association fibers and working memory score. (**c ∼ g**) Node-wise correlation between diffusion metrics of long-range association fibers and working memory scores (**c**: left superior longitudinal fasciculus (SLF) MD; **d:** left inferior fronto-occipital fasciculus RD; **e** left SLF RD; **f**: left inferior longitudinal fasciculus RD; **g**: left arcuate fasciculus RD). All analyses are FDR-corrected (*p* < 0.05) and adjusted for sex and age. Abbreviations: MD, mean diffusivity; RD, radial diffusivity; IFOF, inferior fronto-occipital fasciculus; ILF, inferior longitudinal fasciculus; SLF, superior longitudinal fasciculus; UF, uncinate fasciculus; AF, arcuate fasciculus.

Bundle-wise diffusion metrics also showed particularly robust correlations with working memory, with left IFOF (MD: *r =* -0.7257, *p =* 0.0211; RD: *r =* -0.8315, *p =* 0.0112), right IFOF (RD: *r =* -0.7044, *p =* 0.0295), left ILF (MD: *r =* -0.8111, *p =* 0.0191; RD: *r =* -0.7473, *p =* 0.0243), bilateral SLF (left MD: *r =* -0.6917, *p =* 0.0297, RD: *r =* -0.7562, *p =* 0.0243; right MD: *r =* -0.7412, *p =* 0.0211, RD: *r =* -0.6599, *p =* 0.0391), and bilateral arcuate fasciculus (left MD: *r =* -0.7588, *p =* 0.0211, RD: *r =* -0.7240, *p =* 0.0272; right MD: *r =* -0.7275, *p =* 0.0211, RD: *r =* -0.6835, *p =* 0.0333) reaching significance (FDR-corrected *p <* 0.05, age and sex-adjusted, Fig. 6a and 6b). Node-wise correlation analyses between diffusion metrics and working memory performance revealed significant segments within left SLF (MD, nodes 1–11, 35–63, and 73–100; RD, nodes 1–16 and 80–100), left IFOF (RD, nodes1-4,16–21, and 31–46), left ILF (RD, nodes 1–6), left arcuate fasciculus (RD, nodes1-21, 38–65, and 31–46) (FDR-corrected *p <* 0.05, age and sex-adjusted, Fig. 6c-g). Although these associations survived rigorous multiple comparisons correction and covariate adjustment, the exploratory nature of these analyses in a modest-sized subgroup necessitates cautious interpretation.

## 4 Discussion

The present study systematically characterizes long-range association fiber pathology in anti-NMDAR encephalitis using automated fiber quantification. Three principal discoveries emerged: (i) patients with anti-NMDAR encephalitis exhibited compromised microstructural integrity in major long-range association fibers; (ii) the microstructural damage of long-range association fibers was more pronounced in patients with delayed immunotherapy initiation; and (iii) the microstructural damage of long-range association fibers was substantially correlated with cognitive functions, particularly working memory, in delayed immunotherapy subgroup. This study provides two key advances beyond previous DTI investigations in anti-NMDAR encephalitis. Methodologically, unlike conventional voxel-based approaches(Finke et al., 2013, Liang et al., 2020, Liu et al., 2022, Wang et al., 2024), our tract-specific profiling overcomes the spatial limitations of voxel-based approaches, detecting microstructural alterations with superior sensitivity along complete fiber pathways. Conceptually, by focusing on long-range association fibers rather than deep white matter in prior studies, we reveal their selective vulnerability as critical neural integrators, directly linking structural damage to characteristic cognitive impairments. These findings establish a new framework for understanding neuroinflammation-related long-range connectivity disruption.

The microstructural alterations in long-range association fibers may constitute a distinctive neuroimaging signature of anti-NMDAR encephalitis. The absence of convalescent-phase MRI lesions in our cohort strengthens the interpretation that observed DTI changes reflect primary white matter degeneration rather than residual lesion effects. Our multiparametric DTI analysis revealed complementary roles of MD and RD in characterizing post-inflammatory white matter pathology. Elevated MD reflects generalized microstructural disintegration, capturing extracellular edema, axonal pathology, and myelin breakdown (Bennett et al., 2010). Its sensitivity to both acute and chronic processes positions MD as a global indicator of white matter integrity in convalescent patients (Bennett et al., 2010). Elevated AD reflects axonal damage, while increased RD indicates demyelination (Budde et al., 2007, Klawiter et al., 2011, Klistorner et al., 2022, Song et al., 2005). In the present study, the dissociation between preserved AD and elevated RD further suggests a unique demyelination-predominant pathophysiology in anti-NMDAR encephalitis convalescence. More importantly, RD's stronger correlation with working memory deficits and delayed immunotherapy effects highlights its prognostic value for myelination-dependent cognitive outcomes (Baijot et al., 2022).

In the present study, the impaired microstructural integrity of long-range association fibers likely resulted from underlying demyelination and neuroaxonal degeneration driven by neuroinflammation(Aung et al., 2013). Neuropathological investigations of patients with anti-NMDAR encephalitis have revealed significant inflammatory infiltrates in perivascular and parenchymal regions, particularly within the surrounding white matter (Dalmau et al., 2007, Zrzavy et al., 2021). Our previous study revealed considerably elevated serum neurofilament light chain in convalescent patients with anti-NMDAR encephalitis, suggesting persistent neuroaxonal injury during the recovery phase (Chen et al., 2023). In addition, NMDARs are expressed on developing oligodendrocytes and play a crucial role in myelination (Káradóttir et al., 2005, Salter and Fern, 2005). Neuronal activity orchestrates the differentiation of oligodendrocyte precursor cells and myelination through NMDAR-mediated glutamate signaling(de Rosa et al., 2019, Gautier et al., 2015). Therefore, the observed microstructural damage within the major long-range association in anti-NMDAR encephalitis may be downstream events to the glutamatergic hypofunction, which exacerbates the neuroinflammation-driven demyelination. Moreover, it is important to highlight that long-range association fibers are highly energy-demanding, primarily due to the substantial energy required for action potential conduction along their extensive axons (Ju et al., 2016). The heightened energy demand renders them more susceptible to microstructural damage triggered by neuroinflammation-mediated oxidative stress and ischemic hypoxia (Bercury and Macklin, 2015).

The clinical factors associated with microstructural damage within long-range association fibers were a key focus of our investigation in the present study. Delayed immunotherapy and ICU admission have been widely acknowledged as determinants of poor outcomes in recent systematic reviews(Broadley et al., 2019). Our findings revealed the delayed immunotherapy subgroup showed more pronounced microstructural damage in the long-range association fibers, primarily affecting the cingulum bundles, left uncinate fasciculus, bilateral IFOF, ILF, SLF, and arcuate fascicles. The pathophysiological mechanisms underlying the widespread damage to long-range association fibers in delayed immunotherapy subgroup warrant further exploration. Our recent study employing a humanized mouse model of anti-NMDAR encephalitis revealed neuroinflammation-mediated blood–brain barrier dysfunction occurs early after the onset of neuropsychiatric symptoms (Shu et al., 2023). Chemoattraction of T cells and B cells to the central nervous system predominated during the early stage, triggering antibody-mediated neuroinflammation and immunopathology(Liba et al., 2016). The provoked neuroinflammation typically peaks within the first 2 weeks after disease onset, as evidenced by lymphocytic pleocytosis in cerebrospinal fluid and MRI abnormalities indicative of encephalitis (Dalmau and Graus, 2018). Clinically, patients often develop typical neuropsychiatric symptoms within this 2-weeks window (Dalmau et al., 2019). Therefore, it is plausible that delayed initiation of immunotherapy may exacerbate neuroinflammation-induced microstructural impairments within long-range association fibers, leading to the more widespread damage observed in the delayed subgroup.

Cognitive impairment is a long-term morbidity in patients with anti-NMDAR encephalitis, and delayed treatment has been identified as a predictor of impaired cognitive deficits (Finke et al., 2012, Heine et al., 2021, McKeon et al., 2018). Consistently, the present study revealed a significant correlation between long-range association fiber damage and global cognition in the delayed immunotherapy subgroup. More importantly, our findings revealed a strong correlation between working memory and microstructural damage within these long-range association fibers. Working memory impairment, a core cognitive deficit in patients with anti-NMDAR encephalitis, is attributed to reduced synaptic potentiation caused by NMDAR hypofunction (Stein et al., 2020). The disruption of NMDAR-dependent short-term potentiation serves as the cellular basis of working memory impairment (Fiebig and Lansner, 2017, Kasai et al., 2023). Additionally, NMDAR dysfunction could disrupt the long-range connectivity among different brain regions and hinder the integration of previous and current memories required for working memory storage (Finke et al., 2013, Matute et al., 2020). Our findings suggest that delayed immunotherapy may lead to long-range association fiber damage, thereby contributing to persistent cognitive decline, particularly in the domain of working memory.

Nevertheless, some limitations in the present study should be considered. First, the modest sample size (n = 32 overall, n = 14 in the delayed subgroup), while consistent with the low incidence of anti-NMDAR encephalitis (0.11/100,000 person-years in Asia) (Alentorn et al., 2023) underscores the need for multicenter validation of our findings. Nevertheless, the robust effect sizes surviving FDR correction (all p < 0.05) suggest these preliminary observations merit further investigation in larger cohorts. Second, the interval between disease onset and DTI acquisition varied among patients, with a median interval of 23.4 months (IQR 8.7–43.9) in the delayed immunotherapy subgroup compared to 7.4 months (IQR 4.7–14.1) in the early immunotherapy subgroup. This variability might influence the interpretation of disease progression. Nonetheless, our findings suggest that microstructural damage in long-range association fibers persists over time, independent of the time elapsed since disease onset. Third, this automated fiber quantification pipeline enables precise tract measurements at equidistant nodes, detecting subtle microstructural changes. However, its deterministic tractography struggles with complex fiber configurations, potentially causing inaccuracies. Future work could adopt probabilistic methods to better resolve such architectures. Fourth, while MMSE and MoCA provided feasible global cognition screening, their insensitivity to domain-specific deficits may underestimate true cognitive burden. We intentionally prioritized these scales due to their clinical practicality in convalescent patients with residual disability, but future studies should integrate targeted cognitive assessments.

## 5 Conclusion

In summary, the present study revealed widespread microstructural damage within long-range association fibers among patients with anti-NMDAR encephalitis, suggesting that the timing of immunotherapy initiation may be a critical factor. Substantial damage to long-range association fibers was predominantly observed in patients with delayed immunotherapy initiation and was linked to cognitive performance, with a particular impact on working memory. These findings highlight widespread microstructural alterations in the architecture of long-range association fibers, which may underlie the cognitive impairments observed in this population.

## CRediT authorship contribution statement

**Xiaodong Chen:** Writing – original draft, Visualization, Methodology, Investigation, Funding acquisition, Formal analysis, Data curation. **Ling Fang:** Data curation. **Yiying Huang:** Data curation. **Yu Huang:** Data curation. **Yi Lu:** Data curation. **Jinhui Wang:** Writing – review & editing, Methodology. **Chunxin Liu:** Data curation. **Huanquan Liao:** Writing – review & editing. **Liemin Zhou:** Writing – review & editing. **Wei Qiu:** Writing – review & editing, Supervision, Resources, Project administration, Investigation, Funding acquisition, Conceptualization. **Yaqing Shu:** Writing – review & editing, Supervision, Resources, Project administration, Investigation, Funding acquisition, Conceptualization.

## Funding

This work was supported by grants from the National Natural Science Foundation of China [82271377, 82071343, 82371354, 81701188]; Guangdong Basic and Applied Basic Research Foundation [2022B1515120042, 2021A1515010393]; National Key Research and Development Program of China [2022YFC3600600]; Technology Innovation 2030-Brain Science and Brain-like Research Major Project [2022ZD0208900]; Guangzhou Science and Technology Key R&D Plan [2023B03J1347]; Guangzhou Municipal School (Hospital) Joint Funding [202201020415], First Major Talent Project of the Third Affiliated Hospital of Sun Yat-Sen University (P02099), and Shenzhen Science and Technology Program [JCYJ20230807110700002]. We thank all the patients and healthy controls for volunteering to participate in this study.

## Declaration of competing interest

The authors declare that they have no known competing financial interests or personal relationships that could have appeared to influence the work reported in this paper.

## Data Availability

Data will be made available on request.
