# Supplementary material for: Alterations of long-range association fibers in patients with anti-N-methyl-D-aspartate receptor encephalitis
Source: Neuroimage Clin. 2025 May 24;47:103808. doi: 10.1016/j.nicl.2025.103808 (PMC12158604; doi:10.1016/j.nicl.2025.103808)
Supplement: Supplementary Data 1 [file mmc1.pdf]

Supplementary Materials

Supplementary data for results

Node-wise fractional anisotropy (FA) tract profiles comparison between healthy controls and patient cohort

The result of node-wise FA shows significantly decreased FA in multiple segments of long-range association fibers, including the right cingulum cingulate component (nodes 41-45 and 60-66), left uncinate fasciculus (nodes 28-42), bilateral IFOF (left nodes 1-6, 17-40, 66-74, and 81-87; right nodes 19-39, 85-88, and 97-100), bilateral ILF (left nodes 21-39; right nodes 1-38 and 50-58), and bilateral arcuate fasciculus (left nodes 28-42; right nodes 11-19, age and sex adjusted, FDR-corrected $p < 0.05$; Figure S1a). Principal component analysis (PCA) performed on the mean FA of all long-range association fibers demonstrated that the first three principal components explained 58.69% of the total variance (Figure S1b).

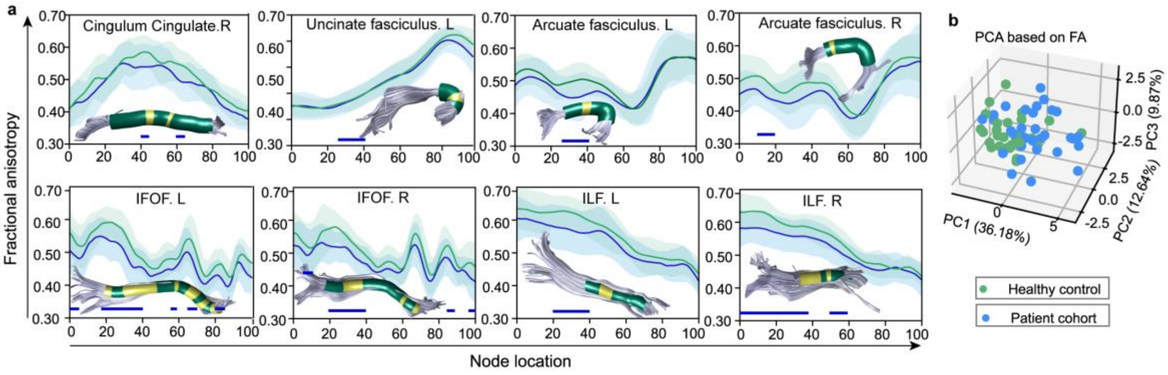

Figure S1 Group difference of node-wise fractional anisotropy (FA) tract profiles of major long association fibers and principal component analysis (PCA). **(a)** Node-wise FA tract profiles between two groups comparisons (FDR-corrected $p < 0.05$, adjusted for sex and age). The blue segments indicate subregions with statistically significant differences between groups. **(b)** PCA based on the mean FA of all long-range association fibers. Abbreviations: FA, fractional anisotropy; PC, principal component

Correlation analyses between diffusion metrics of long-range association fibers and the time to immunotherapy commencement

Interestingly, the time to immunotherapy commencement exhibited a significant linear positive correlation with the diffusion metrics of several long-range association fibers, including the right IFOF (MD: r = 0.3667, p = 0.0462), left ILF (MD: r = 0.4119, p = 0.0237), left uncinate fasciculus (RD: r = 0.4486, p = 0.0129; MD: r = 0.3841, p = 0.0361), and right arcuate fasciculus (RD: r = 0.4447, p = 0.0138; MD: r = 0.4161, p = 0.0222), after adjusting for sex and age (Supplementary Figure S2).

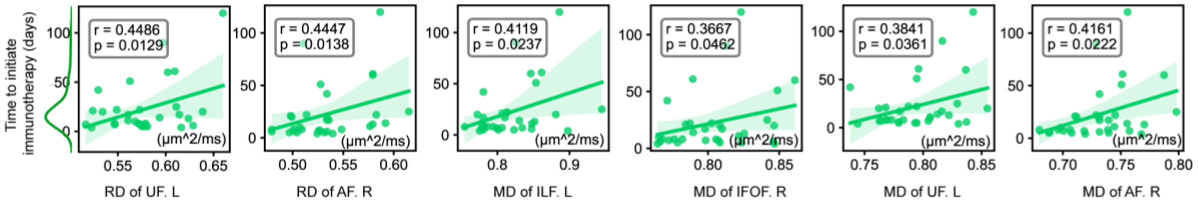

Figure S2 Linear correlation between the time to immunotherapy initiation(days) and the mean diffusivity and radial diffusivity of long-range association fibers (adjusted for sex and age). Abbreviations: MD, mean diffusivity; RD, radial diffusivity; IFOF, inferior fronto-occipital fasciculus; ILF, inferior longitudinal fasciculus; UF, uncinate fasciculus; AF, arcuate fasciculus

Supplementary Tables

Table S1 Summary of the major long-range association fibers in the brain

Long-range association fiber groups	Abbreviations
Cingulum cingulate component	CC
Cingulum hippocampal component	CHip
Inferior fronto-occipital fasciculus	IFOF
Inferior longitudinal fasciculus	ILF
Superior longitudinal fasciculus	SLF
Uncinate fasciculus	UF
Arcuate fasciculus	AF

Table S2 Group difference of diffusion features between healthy controls and patient cohort at bundle-wise level

Diffusion metrics	Patient cohort N=32	Healthy controls N=30	*F* value	$p^{\dagger}$ *FDR-corrected*
Mean fractional anisotropy				
CC. R	0.4812 ± 0.0412	0.5035 ± 0.0331	5.7506	0.0394
CHip. R	0.4182 ± 0.0412	0.4466 ± 0.0336	7.7617	0.0189
IFOF. L	0.4673 ± 0.0329	0.5024 ± 0.0286	21.7287	0.0003
IFOF. R	0.4773 ± 0.0286	0.5057 ± 0.0322	13.7887	0.0017
ILF. L	0.4286 ± 0.0325	0.4569 ± 0.0258	13.7484	0.0017
ILF. R	0.4165 ± 0.0323	0.4431 ± 0.0219	13.7328	0.0017
AF. R	0.4561 ± 0.0350	0.4813 ± 0.0288	7.5202	0.0189
Mean mean diffusivity, μm^2/ms				
CC. R	0.7610 ± 0.0259	0.7426 ± 0.0301	5.9981	0.0383
CHip. L	0.7939 ± 0.0285	0.7767 ± 0.0278	4.9953	0.0456
IFOF. L	0.7990± 0.0309	0.7799 ± 0.0202	8.4558	0.0281
ILF. L	0.8201 ± 0.0423	0.7936 ± 0.0245	8.1313	0.0281
ILF. R	0.8153 ± 0.0349	0.7954 ± 0.0246	5.7488	0.0383
SLF. L	0.7268 ± 0.0332	0.7088 ± 0.0242	5.5474	0.0383
UF. L	0.7952 ± 0.0288	0.7739 ± 0.0195	9.5064	0.0281
AF. L	0.7354 ± 0.0351	0.7161 ± 0.0227	6.8584	0.0383
AF. R	0.7316 ± 0.0283	0.7144 ± 0.0237	6.2988	0.0383
Mean radial diffusivity, μm^2/ms				
CC. R	0.5383 ± 0.0369	0.5117 ± 0.0370	7.8006	0.0161
CHip. R	0.5920 ± 0.0435	0.5637 ± 0.0294	7.2615	0.0161
IFOF. L	0.5725 ± 0.0371	0.5369 ± 0.0237	22.0444	0.0002
IFOF. R	0.5713 ± 0.0289	0.5444 ± 0.0329	11.8968	0.0043
ILF. L	0.6131 ± 0.0457	0.5760 ± 0.0262	14.3341	0.0026
ILF. R	0.6186 ± 0.0387	0.5878 ± 0.0279	11.5474	0.0043
SLF. R	0.5252 ± 0.0347	0.4992 ± 0.0342	7.3808	0.0161
UF. L	0.5781 ± 0.0355	0.5586 ± 0.0259	4.7336	0.0471
AF. L	0.5278 ± 0.0436	0.5042 ± 0.0284	5.9222	0.0281
AF. R	0.5339 ± 0.0353	0.5075 ± 0.0297	8.5905	0.0135

†: *p* value of analysis of covariance (ANCOVA) after adjusting for age, sex and FDR correction Abbreviations: CC, cingulum cingulate component; CHip, cingulum hippocampal component; IFOF, inferior fronto-occipital fasciculus; ILF, inferior longitudinal fasciculus; SLF, superior longitudinal fasciculus; UF, uncinate fasciculus; AF, arcuate fasciculus

Table S3 Loadings of PCA based on bundle-wise FA

Diffusion features	Loadings		
	PC 1	PC 2	PC 3
FA-CC.L	-0.2032	-0.5405	-0.1408
FA-CC.R	-0.2754	-0.4391	0.0685
FA-CHip.L	-0.2372	0.2915	0.2905
FA-CHip.R	-0.2309	0.3960	-0.1223
FA-IFOF.L	-0.2958	0.1041	-0.4039
FA-IFOF.R	-0.3326	-0.0221	-0.0816
FA-ILF.L	-0.3048	0.0518	-0.1712
FA-ILF.R	-0.3470	-0.0438	-0.1166
FA-SLF.L	-0.0972	-0.3334	-0.2313
FA-SLF.R	-0.3086	-0.0900	0.1110
FA-UF.L	-0.2057	0.0617	0.4481
FA-UF.R	-0.1682	-0.1979	0.6199
FA-AF.L	-0.3259	0.1605	0.0736
FA-AF.R	-0.2834	0.2633	-0.0843

Abbreviations: PCA, principal component analysis; FA, fractional anisotropy; PC, principal component; CC, cingulum cingulate component; CHip, cingulum hippocampal component; IFOF, inferior fronto-occipital fasciculus; ILF, inferior longitudinal fasciculus; SLF, superior longitudinal fasciculus; UF, uncinate fasciculus; AF, arcuate fasciculus

Table S4 Loadings of PCA based on bundle-wise MD

Diffusion features	Loadings		
	PC 1	PC 2	PC 3
MD-CC.L	0.2528	-0.0245	-0.4554
MD-CC.R	0.2766	0.0404	-0.3188
MD-CHip.L	0.1787	-0.1812	0.7697
MD-CHip.R	0.1616	-0.6264	-0.2072
MD-IFOF.L	0.2938	0.0295	-0.0848
MD-IFOF.R	0.2706	-0.0628	0.0367
MD-ILF.L	0.2996	0.0398	-0.0750
MD-ILF.R	0.3009	-0.0684	0.0669
MD-SLF.L	0.2856	0.2820	0.0314
MD-SLF.R	0.2931	0.2605	0.0580
MD-UF.L	0.2662	-0.2882	0.0351
MD-UF.R	0.2290	-0.4176	0.1088
MD-AF.L	0.2992	0.2337	0.0763
MD-AF.R	0.2853	0.3195	0.1176

Abbreviations: PCA, principal component analysis; MD, mean diffusivity; PC, principal component；CC, cingulum cingulate component; CHip, cingulum hippocampal component; IFOF, inferior fronto-occipital fasciculus; ILF, inferior longitudinal fasciculus; SLF, superior longitudinal fasciculus; UF, uncinate fasciculus; AF, arcuate fasciculus

Table S5 Loadings of PCA based on bundle-wise RD

Diffusion features	Loadings		
	PC 1	PC 2	PC 3
RD-CC.L	0.2339	0.2964	-0.4672
RD-CC.R	0.2752	0.2514	-0.3981
RD-CHip.L	0.2049	-0.3713	0.2173
RD-CHip.R	0.1714	-0.5241	0.1024
RD-IFOF.L	0.2899	0.0953	0.2178
RD-IFOF.R	0.2853	-0.0325	-0.0852
RD-ILF.L	0.3118	-0.0332	0.1374
RD-ILF.R	0.3170	-0.0746	-0.0285
RD-SLF.L	0.2582	0.3833	0.1034
RD-SLF.R	0.2934	0.1423	0.1102
RD-UF.L	0.2564	-0.3517	-0.2237
RD-UF.R	0.2045	-0.3290	-0.4738
RD-AF.L	0.3078	0.1012	0.2501
RD-AF.R	0.2828	0.1010	0.3622

Abbreviations: PCA, principal component analysis; RD, radial diffusivity; PC, principal component; CC, cingulum cingulate component; CHip, cingulum hippocampal component; IFOF, inferior fronto-occipital fasciculus; ILF, inferior longitudinal fasciculus; SLF, superior longitudinal fasciculus; UF, uncinate fasciculus; AF, arcuate fasciculus

Table S6 MD and RD difference among healthy controls and patient subgroups at bundle-wise level

Diffusion features	Healthy controls (N=30)	Early immunotherapy subgroup(N=18)	Delayed immunotherapy subgroup(N=14)	*F* value	*p* value	Post-hoc†	Post-hoc‡	FDR-corrected *p*¶	FDR-corrected *p*§
Mean mean diffusivity, μm^2/ms									
CC. R	0.7426 ± 0.0306	0.7556 ± 0.0226	0.7679 ± 0.0298	3.799	0.028	**0.0237**	0.3764	0.0814	0.5147
IFOF. L	0.7799 ± 0.0206	0.7890 ± 0.0277	0.8119 ± 0.0320	7.128	0.002	**0.0010**	0.4450	**0.0220**	0.5147
ILF.L	0.7936 ± 0.0249	0.8093 ± 0.0363	0.8340 ± 0.0482	6.313	0.003	**0.0022**	0.4020	**0.0220**	0.5147
ILF.R	0.7954 ± 0.0250	0.8080 ± 0.0346	0.8246 ± 0.0355	4.300	0.018	**0.0134**	0.5178	0.0643	0.5592
SLF.L	0.7088 ± 0.0247	0.7216 ± 0.0313	0.7336 ± 0.0366	3.354	0.042	**0.0377**	0.3705	0.1130	0.5147
UF. L	0.7739 ± 0.0199	0.7906 ± 0.0255	0.8012 ± 0.0334	6.044	0.004	**0.0037**	0.2041	**0.0220**	0.4081
AF. L	0.7161 ± 0.0231	0.7284 ± 0.0311	0.7443 ± 0.0402	4.193	0.020	**0.0175**	0.2978	0.0701	0.4765
AF. R	0.7227 ± 0.0289	0.7144 ± 0.0241	0.7431 ± 0.0250	5.740	0.005	**0.0037**	0.6263	**0.0220**	0.6263
Mean radial diffusivity, μm^2/ms									
CC. R	0.5117 ± 0.0370	0.5300 ± 0.0295	0.5490 ± 0.0434	5.394	0.007	**0.0053**	0.3363	**0.0252**	0.5064
CHip.R	0.5637 ± 0.0294	0.6010 ± 0.0441	0.5804 ± 0.0413	4.390	0.017	0.3376	**0.0137**	0.5064	**0.0457**
IFOF. L	0.5369 ± 0.0237	0.5646 ±0.0296	0.5827 ± 0.0440	11.875	0.000	**0.0000**	**0.0059**	**0.0030**	**0.0252**
IFOF.R	0.5444 ± 0.0329	0.5683 ± 0.0314	0.5752 ± 0.0259	5.959	0.004	**0.0114**	**0.0316**	**0.0427**	0.0947
ILF.L	0.5760 ± 0.0262	0.6055 ± 0.0361	0.6228 ± 0.0556	8.081	0.0008	**0.0010**	**0.0467**	**0.0147**	0.1078
ILF. R	0.5878 ± 0.0279	0.6148 ± 0.0405	0.6236 ± 0.0371	6.131	0.004	**0.0058**	0.0606	**0.0252**	0.1300
SLF. R	0.4992 ± 0.0341	0.5180 ± 0.0414	0.5345 ± 0.0215	5.318	0.008	**0.0055**	0.3900	**0.0252**	0.5225
UF. L	0.5586 ± 0.0259	0.5741 ± 0.0302	0.5833 ± 0.0421	3.192	0.049	**0.0394**	0.5148	0.0984	0.5720
AF. L	0.5042 ± 0.0284	0.5233 ± 0.0409	0.5348 ± 0.0474	3.462	0.038	**0.0363**	0.3153	0.0984	0.5064
AF. R	0.5075 ± 0.0297	0.5254 ± 0.0326	0.5450 ± 0.0368	6.642	0.003	**0.0017**	0.3754	**0.0168**	0.5225

Abbreviations: CC, cingulum cingulate component; IFOF, inferior fronto-occipital fasciculus; ILF, inferior longitudinal fasciculus; SLF, superior longitudinal fasciculus; UF, uncinate fasciculus; AF, arcuate fasciculus

†: post-hoc comparison between healthy controls and delayed immunotherapy subgroup; ‡: post-hoc comparison between healthy controls and early immunotherapy subgroup; ¶: FDR-corrected *p* values for post-hoc comparison between healthy controls and delayed immunotherapy; §: FDR-corrected *p* values for post-hoc comparison between healthy controls and early immunotherapy subgroup
